# Hypoxia-Induced Fibroblast Growth Factor 11 Stimulates Osteoclast-Mediated Resorption of Bone

**DOI:** 10.1007/s00223-016-0228-1

**Published:** 2017-01-18

**Authors:** Helen J. Knowles

**Affiliations:** Botnar Research Centre, Nuffield Department of Orthopaedics Rheumatology & Musculoskeletal Sciences, University of Oxford, Headington, Oxford, OX3 7LD UK

**Keywords:** Osteoclast, Hypoxia-inducible factor-1 alpha (HIF-1α), Bone resorption, Microtubule, Rheumatoid arthritis

## Abstract

Over-activation of osteoclasts is directly responsible for pathological bone loss in conditions such as rheumatoid arthritis and cancer metastasis to bone. Hypoxia is a common feature of these conditions, associated with poor prognosis, which also stimulates osteoclast-mediated bone resorption via induction of the hypoxia-inducible transcription factor HIF-1α. Here, we investigate the effects of fibroblast growth factor 11 (FGF11) on osteoclast function. FGF11 is an intracellular FGF that was induced both by hypoxia (2% O_2_, *p* < 0.01) and by inhibition of the HIF-regulating prolyl hydroxylase enzymes (CoCl_2_, *p* < 0.001) in osteoclasts. Isoform-specific siRNA demonstrated that the induction of *Fgf11* mRNA expression by hypoxia is HIF-1α-dependent (*p* < 0.01). Hypoxic stimulation of bone resorption was inhibited in osteoclasts treated with siRNA targeting FGF11 (*p* < 0.05). This was at least partially due to reduced secretion of an unidentified pro-resorptive factor downstream of FGF11. FGF11 expression within hypoxic, resorbing osteoclasts co-localised with microtubule-associated alpha-tubulin. FGF11 was also abundantly expressed in osteoclasts within the rheumatoid synovium and in giant cell tumour of bone. This study suggests FGF11 as a novel factor driving pathological bone resorption in osteolytic disease and as a potential target for the development of new anti-resorptive therapeutic agents.

## Introduction

A balance between bone formation, performed by osteoblasts, and bone resorption, performed by osteoclasts, regulates developmental bone remodelling and maintains bone integrity throughout life. Pathological bone loss occurs when this homeostatic relationship is disturbed. Over-activation of osteoclasts is directly responsible for the resorptive bone loss evident in diseases such as rheumatoid arthritis [1, 2], cancer metastasis to bone [3] and osteoporosis [4].

Micro-environmental hypoxia is a common feature of these osteolytic conditions, in all of which hypoxia and / or stabilisation of hypoxia-inducible factor (HIF) correlates with disease progression and poor prognosis [5–7]. HIF is a heterodimeric transcription factor, the stability and transcriptional activity of which is controlled via regulation of the hypoxia-inducible alpha subunits (HIF-1α, HIF-2α). In normoxic conditions HIF-α is post-translationally hydroxylated by a set of three oxygen-dependent prolyl hydroxylase domain enzymes (PHD1-3), targeting it for proteasomal degradation. An additional regulatory enzyme, asparagine hydroxylase factor inhibiting HIF (FIH), inhibits the transcriptional activity of any remaining HIF. Activity of the HIF hydroxylases is limited by oxygen deficiency, allowing hypoxic stabilisation of HIF-α and binding of the active HIF complex to the hypoxia-response element (HRE) of HIF target genes [8].

Hypoxia causes a HIF-1α-dependent increase in the ability of in vitro cultured osteoclasts to resorb bone [9–11]. Using osteoclast-specific conditional HIF-1α knockout mice, it was also shown that HIF-1α is essential for driving the resorptive bone loss associated with the osteoporotic phenotype in ovariectomised mice [7]. To identify the genes and pathways driving the hypoxic increase in bone resorption, we previously performed a microarray comparing gene expression in normoxic versus hypoxic (24 h, 2% O_2_) osteoclasts [11]. This led to the identification of pro-resorptive effects for the adipocytokine angiopoietin-like 4 (ANGPTL4) [11, 12] and for the metabolic enzymes regulating hypoxic production of ATP [13].

Another gene of interest from the microarray was fibroblast growth factor 11 (FGF11), also known as FGF homologous factor 3 (FHF3), which belongs to the family of intracellular non-secreted FGFs that includes FGF12 (FHF1), FGF13 (FHF2) and FGF14 (FHF4). These intracellular FGFs do not bind either the classical FGF receptors or heparan sulphate glycosaminoglycans and are thought to bind target proteins distinct from those of the classical FGFs [14]. While cytoplasmic binding partners have been suggested for the other FHFs, including the MAPK scaffold protein islet-brain 2 (IB2) [15], voltage-gated sodium channels [16, 17] and cardiac calcium channels [18], no interaction partner has been described for FGF11.

Similarly, little is known about the function of FGF11. *Fgf11* mRNA expression was described in the epithelial signalling centres and epithelial-mesenchymal interfaces at key stages of mouse tooth formation, suggestive of a role in odontogenesis [19]. *Fgf11* mRNA was also elevated during a full-thickness mouse skin wound healing experiment [20] and in fibroblasts seeded in vitro into a fibrous hydrogel scaffold to support skin repair [21]. Additionally, FGF11 over-expression in endothelial cells stimulated capillary-like tube formation in vitro [22]. A developmental or tissue remodelling role for FGF11 therefore seems likely. This study aims to investigate the mechanism of induction of FGF11 in hypoxic osteoclasts and to identify any effects of FGF11 on the regulation of osteoclast-mediated resorption of bone.

## Materials and Methods

### Human Osteoclast Culture

Peripheral blood mononuclear cells (PBMCs) were isolated from leucocyte cones using Histopaque (Sigma). Positively selected CD14+ monocytes were seeded onto tissue culture plates or dentine slices in α-MEM culture media (without ribonucleosides/deoxyribonucleosides) containing 10% FBS, L-glutamine (2 mM), penicillin (50 IU/ml) and streptomycin sulphate (50 μg/ml). Cultures were supplemented with M-CSF (25 ng/ml) + RANKL (35 ng/ml) every 3–4 days, with mature osteoclasts being formed by day 9. Antibodies were against the vitronectin receptor (VNR) (CD51/61 monoclonal antibody, AbD Serotec) and alpha-tubulin (4a, GeneTex). F-actin was visualised using TRITC-phalloidin. Multinucleated cells containing ≥3 nuclei were considered osteoclasts. Resorption pits were stained with 0.5% toluidine blue, photographed and quantified using ImageJ.

### Cell Culture

THP1 (monocytic leukaemia), Jurkat (T cell leukaemia), MG-63 and Saos2 (osteoblastic osteosarcoma) cell lines were maintained in RPMI 1640 supplemented with 10% FBS, L-glutamine (2 mM), penicillin (50 IU/ml) and streptomycin sulphate (50 μg/ml). Primary human CD14+ monocytes were separated from PBMCs and maintained in αMEM supplemented with 25 ng/ml M-CSF. CD4+ T cells were positively selected from the same PBMCs. Primary human osteoblasts were obtained by outgrowth from cancellous bone chips removed during surgery. Mononuclear GCTB stromal cells were isolated from GCTB as described previously [23]. Mouse bone marrow cells were flushed from the long bones of C57BL/6 wild-type and PHD2^+/−^ mice [24] and cultured in αMEM supplemented with 25 ng/ml M-CSF for 2 days. Hypoxic exposure (2% O_2_, 5% CO_2_, balance N_2_) was performed in a MiniGalaxy incubator.

### Real-Time PCR

RNA was extracted in TRI reagent then DNase-treated and eluted using the Direct-Zol RNA Miniprep kit (Zymo Research). RNA was reverse-transcribed and then real-time PCR was performed with Fast SYBR Green Master Mix (Applied Biosystems) and human QuantiTect primers (Qiagen). Primers were designed in-house to murine *Fgf11*; S: 5′-CCAGCTCCTTCACCCACTTC-3′, AS: 5′-CAATAGCCCCTCAGCGTTCA-3′ and murine *Actb*; S: 5′-ATGTGGATCAGCAAGCAGGAG-3′, AS: 5′-GTGTAAAACGCAGCTCAGTAACA-3′. Comparative quantification was performed, with target gene expression normalized to β-actin (*Actb*).

### Western Blot Analysis and ELISA

Cells were homogenized in lysis buffer (6.2 M urea, 10% glycerol, 5 mM DTT, 1% SDS, protease inhibitors). Primary antibodies were against FGF11 (ab129375, Abcam), HIF-1α (clone 54; BD Biosciences), MMP9 (EP1254, Abcam) and β-tubulin (TUB2.1, Sigma). Images were either scanned from X-ray film or acquired using the Uvitec Alliance imaging system and NineAlliance software. Densitometry was performed in ImageJ and normalized to the β-tubulin signal. ELISA kits were against FGF11 (USCNK) and ANGPTL4 (R&D Systems).

### Plasmid Cloning and siRNA Transfection

Mature osteoclasts were transfected with 50 nM siRNA targeting HIF-1α or a HIF-1α scrambled control [25] or with Silencer Select Pre-designed siRNA targeting FGF11 (s5152 [F2] and s5153 [F3]) or a negative control (Ambion) using RNAiMAX (Invitrogen). Duplexes were removed after 4 h and osteoclasts incubated in osteoclast differentiation media. Human FGF11 (TrueClone Full-Length cDNA, OriGene) was cloned into pcDNA3 using EcoRI and NotI. The FGF11-pcDNA3 expression construct was transfected into MG-63 cells using Lipofectamine 2000 (Invitrogen).

### Metabolic Assays

Glucose was measured in cell culture medium using the Glucose (GO) Assay Kit (Sigma). Mitochondrial dehydrogenase activity was assessed using Alamar blue (AbD Serotec). All results were normalized to cell number determined using crystal violet, which stains nuclei independently of cellular metabolic status, which was then extracted and absorbance read at 550 nm.

### Immunohistochemistry

Human tissue sections were obtained from the Oxford Musculoskeletal Biobank (OMB); the study was approved under OMB ethics (HTA Licence 12217, Oxfordshire Research Ethics Committee C, 09/H0606/11) and carried out in accordance with the associated guidelines and regulations. Informed consent was obtained from all subjects. GCTB tissue (*n* = 5 tumours) and RA tissue (*n* = 3 samples) was from samples previously identified as containing HIF-positive osteoclasts [12, 26]. Antigen retrieval of deparaffinised formalin-fixed tissue sections was performed by microwaving in 1 mM EDTA (pH 8). Sections were exposed to anti-FGF11 antibody (ab129375, Abcam) with normal rabbit serum as a negative control. Staining was visualised with the VECTASTAIN Elite ABC Kit (Vector Laboratories). Osteoclasts in tissue sections were classified as large, multinucleated cells containing  ≥3 nuclei.

### Statistics

Results are represented as mean ± standard error of at least *n* = 3 independent experiments. Statistical analysis comprises one-way or two-way ANOVA using Dunnett’s comparison as a post hoc test. For experiments with only two conditions, a Students’s *t* test was applied. Results were considered significant at values of *p* < 0.05.

## Results

### FGF11 Expression is Induced by Hypoxia

**Fig. 1:**
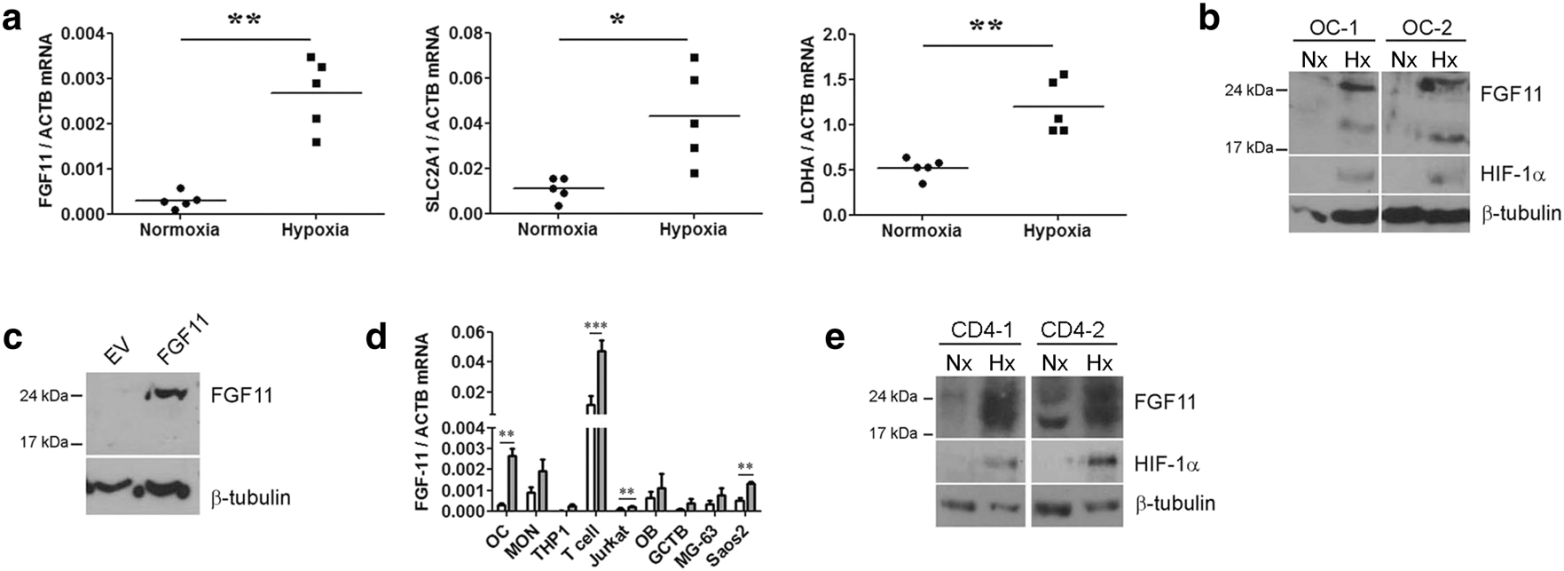
**a** Comparative quantitation of *Fgf11, Slc2a1* and *Ldha* mRNA in mature human osteoclasts following 24 h exposure to normoxia or hypoxia (2% O_2_) (*Slc2a1* = solute carrier family 2 member 1/glucose transporter 1 (*Glut-1*), *Ldha* = lactate dehydrogenase A, *Actb* = β-actin). *p < 0.05; **p < 0.01. **b** Expression of HIF-1α and FGF11 protein in two independent osteoclast populations (OC-1, OC-2) after 24 h exposure to normoxia (Nx) or hypoxia (2% O_2_, Hx). **c** Expression of FGF11 protein in MG-63 cells 72 h after transfection with FGF11-pcDNA3 (FGF-11) or an empty vector (EV) control. **d** Comparative quantitation of *Fgf11* mRNA in multiple cell types (OC, primary osteoclasts; MON, CD14+ monocytes; THP1, monocytic cell line; T cell, CD4+ T cell; Jurkat, T cell line; OB, primary human osteoblasts; GCTB, stromal cells from Giant Cell Tumour of Bone, MG-63 and Saos2, osteoblastic cell lines) following 24 h exposure to normoxia or hypoxia (2% O_2_). **p > 0.01; ***p < 0.001. **e** Expression of FGF11 protein in two independent CD4+ T cell populations (CD4-1, CD4-2) following 24 h exposure to normoxia (Nx) or hypoxia (2% O_2_, Hx)

*Fgf11* (NM_004112.2) exhibited 4.7-fold hypoxic induction (*p* < 0.00001) in our published microarray comparing gene expression in normoxic versus hypoxic (24 h, 2% O_2_) osteoclasts [11]. Induction of *Fgf11* by 24 h exposure to 2% O_2_ was confirmed in in vitro differentiated osteoclasts by real-time PCR (Fig. 1a), hypoxic culture conditions being confirmed by parallel induction of two established hypoxia-regulated genes (*Slc2a1, Ldha*).

Hypoxic induction of FGF11 protein was observed by Western blot after 24 h exposure to 2% O_2_ (Fig. 1b), with hypoxic culture conditions being confirmed by stabilisation of HIF-1α protein.

Densitometry indicated a hypoxic induction of 3.36-fold ± 0.81 (*p* < 0.05) for FGF11 protein. Hypoxia-inducible bands were detected with molecular weights of approximately 20 and 24 kDa. Transfection of a full-length human FGF11 construct into MG-63 cells produced FGF11 protein running at 24 kDa, suggesting that the 20 kDa form could be the result of alternative post-translational processing of FGF11 in osteoclasts (Fig. 1c). No FGF11 was detected in the conditioned media of either hypoxic osteoclasts or MG-63 transfectants by either Western blot or ELISA, consistent with FGF11 being an intracellular FGF.

To compare levels of expression of *Fgf11* mRNA in other bone-related cell types, we exposed primary osteoclasts, monocytes, T cells and osteoblasts, as well as associated cell lines, to normoxia and hypoxia for 24 h (Fig. 1d). Primary cells showed much higher levels of *Fgf11* mRNA than cell lines of the same origin. Osteoclasts and the CD14+ monocytes from which they derive expressed similar levels of *Fgf11*. However, CD4+ T cells had the highest expression of *Fgf11* in both normoxic and hypoxic conditions. Normoxic expression of FGF11 protein was confirmed in CD4+ T cells by Western blot, as well as a striking hypoxic induction (Fig. 1e).

### Hypoxic Induction of FGF11 is Regulated by HIF-1α

Yang et al. recently described HIF-1α-dependent activation of two HREs in the FGF11 promoter, using co-transfection of promoter luciferase constructs and HIF-1α over-expression plasmids into endothelial cells (HUVEC) [22]. We therefore investigated whether hypoxic induction of FGF11 was also HIF-1α-dependent in osteoclasts.

**Fig. 2:**
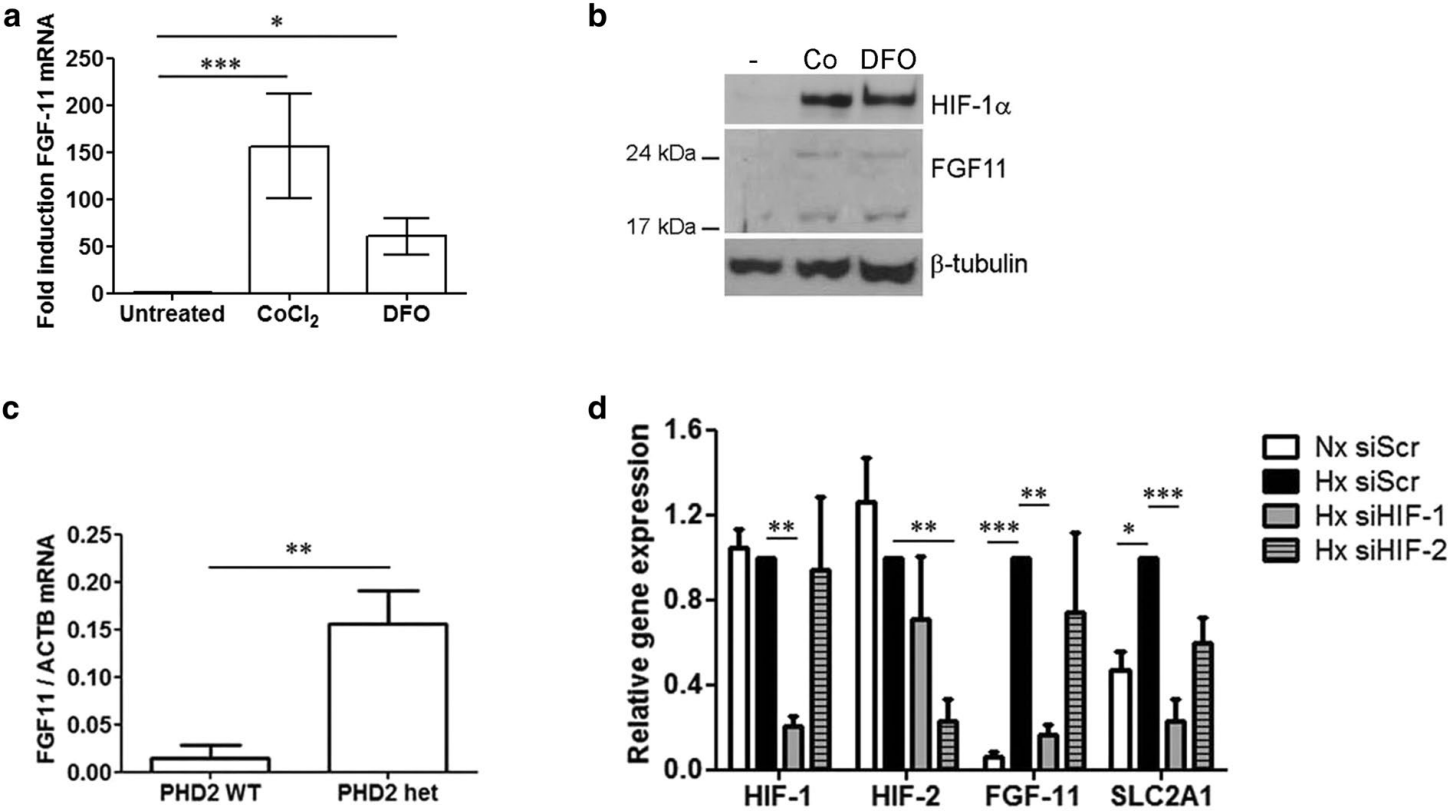
Comparative expression of **a** *Fgf11* mRNA and **b** FGF11 protein in mature human osteoclasts following 24 h exposure to CoCl_2_ (100 μM) or desferrioxamine (DFO, 100 μM). **c** Comparative quantitation of *Fgf11* mRNA in PHD2 wild-type and PHD2^+/−^ bone marrow cells. **d** Effect of siRNA targeting HIF-1α or HIF-2α, or a HIF-1α scrambled control (scr), on expression of *HIF-1α, HIF-2α, Slc2a1* or *Fgf11* mRNA. *p < 0.05; **p > 0.01; ***p < 0.001

Normoxic stabilisation of HIF-1α by CoCl_2_ or desferrioxamine (DFO) increased expression of *Fgf11* mRNA (Fig. 2a) and FGF11 protein (Fig. 2b) in primary human osteoclasts. As neither stimulus specifically inhibits the HIF-regulating PHD enzymes, we also investigated effects of genetic inactivation of PHD2, the primary regulator of HIF instability in normoxia [27]. *Fgf11* mRNA expression was elevated in the bone marrow cells of PHD2^+/−^ mice in comparison with wild-type littermate controls (Fig. 2c). Treatment of osteoclasts with isoform-specific siRNA targeting either HIF-1α or HIF-2α revealed that hypoxic induction of *Fgf11* mRNA is regulated by HIF-1α, in a similar manner to the known HIF-1α target gene *Slc2a1* (Fig. 2d).

### FGF11 Mediates Hypoxic Induction of Osteoclast-Mediated Bone Resorption

**Fig. 3:**
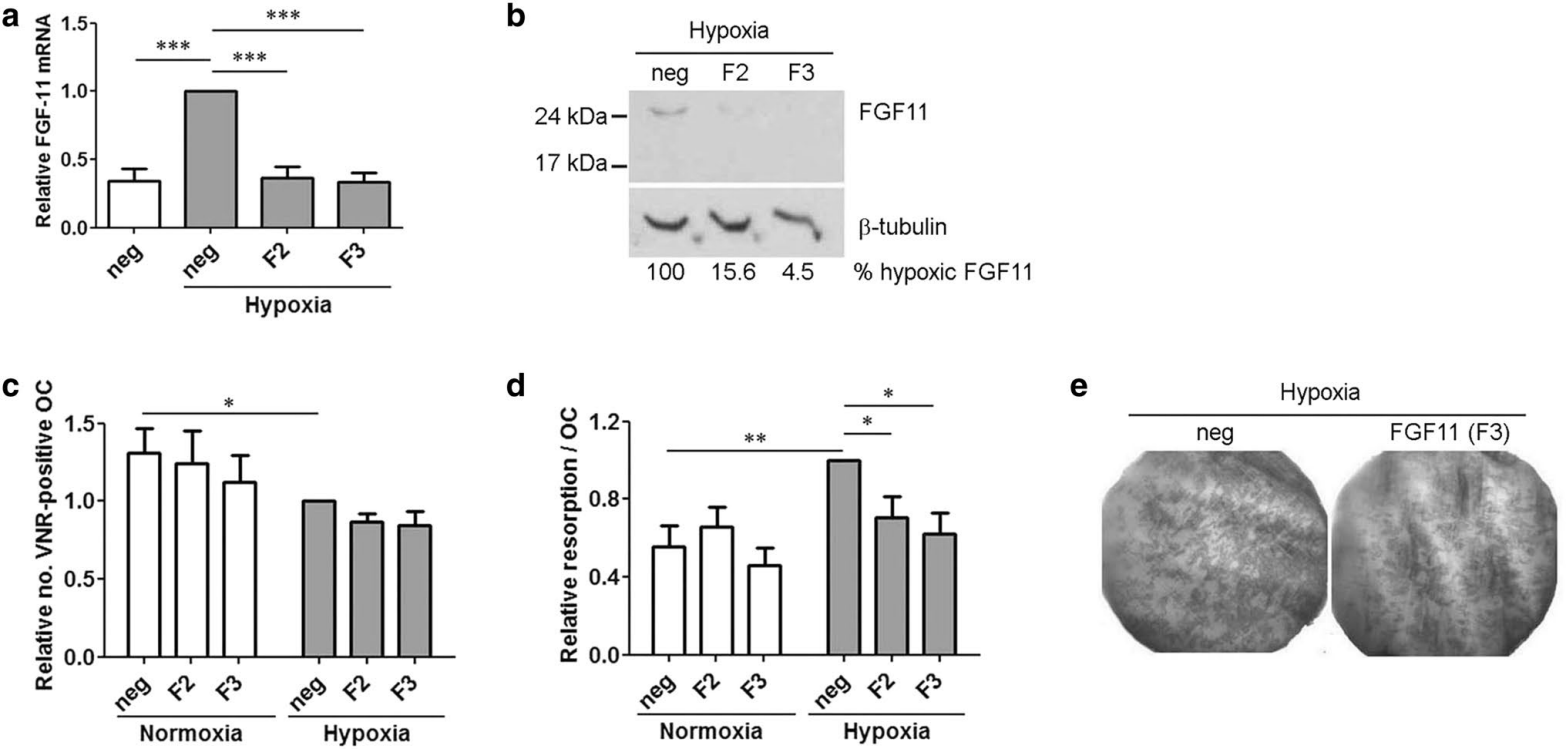
Comparative quantitation of **a** *Fgf11* mRNA and **b** FGF11 protein in mature human osteoclasts treated with siRNA targeting FGF11 (F2, F3) or a negative control siRNA (neg) followed by 24 h exposure to hypoxia (2% O_2_). Densitometry was used to quantify percentage reduction in hypoxic FGF11 protein due to siRNA treatment. **c** Quantification of the number of VNR-positive, multinucleated osteoclasts and **d** quantified resorption (resorption track area per osteoclast) following treatment with FGF11 or negative control siRNA and 24 h exposure to either normoxia or hypoxia (2% O_2_). **e** Representative dentine slices showing toluidine blue-stained resorption tracks. *p < 0.05; **p > 0.01; ***p < 0.001

To determine whether FGF11 affects osteoclast function, mature osteoclasts were transfected with two independent siRNAs targeting FGF11 (F2 and F3) or a negative control siRNA (neg). Both FGF11 siRNAs completely ablated the hypoxic increase in *Fgf11* mRNA (Fig. 3a) and dramatically reduced hypoxic induction of FGF11 protein (Fig. 3b). FGF11 siRNA did not significantly affect osteoclast number in either normoxic or hypoxic (24 h, 2% O_2_) conditions (Fig. 3c). However, knock-down of FGF11 did reduce the hypoxic increase in osteoclast-mediated bone resorption by 73.8% (F2 siRNA) and 82.1% (F3 siRNA), respectively, (Fig. 3d, e), without affecting normoxic levels of resorption.

### Mechanism of Action for FGF11-Mediated Bone Resorption

**Fig. 4:**
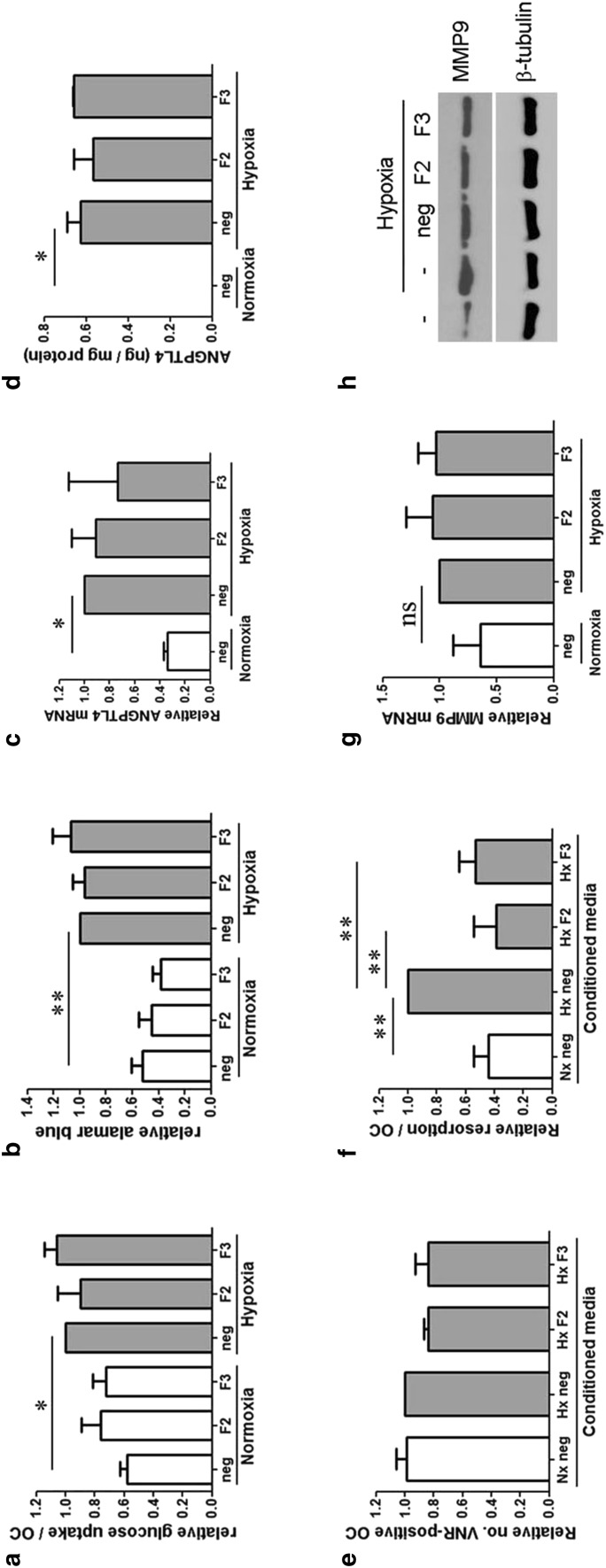
**a** Relative glucose consumption and **b** relative Alamar Blue fluorescence following 24 h culture in normoxia or hypoxia (2% O_2_). Results are normalized to cell number and expressed relative to the hypoxic control. **c** Comparative quantitation of *Angptl4* mRNA and **d** ANGPTL4 protein in mature human osteoclasts treated with siRNA targeting FGF11 (F2, F3) or a negative control siRNA (neg) followed by 24 h exposure to either normoxia or hypoxia (2% O_2_). **e, f** Effect of conditioned media from osteoclasts treated with FGF11 siRNA and exposed to normoxia (Nx) or hypoxia (Hx) for 24 h on **e** the number of VNR-positive, multinucleated osteoclasts and **f** bone resorption (resorption track area per osteoclast). **g** Comparative quantitation of *Mmp9* mRNA and **h** MMP9 protein in mature human osteoclasts treated with FGF11 siRNA. *p < 0.05; **p > 0.01

As there are no known interaction partners for FGF11, we next investigated whether FGF11 modulates pathways already known to mediate the hypoxic induction of bone resorption by osteoclasts. However, FGF11 siRNA did not affect the hypoxic increase in either glucose consumption (Fig. 4a) or mitochondrial reductase activity (Fig. 4b) observed in osteoclasts. Neither did it affect hypoxic expression of the pro-resorptive adipokine ANGPTL4 (Fig. 4c, d).

To determine whether effects of FGF11 were mediated by an intracellular or secreted factor, we collected conditioned media from osteoclasts treated with FGF11 siRNA and subsequently exposed to normoxic or hypoxic conditions for 24 h. This conditioned media was then applied to fresh mature osteoclasts for a further 24 h. No conditioned media had any effect on osteoclast number (Fig. 4e), but hypoxic control media caused a twofold increase in bone resorption that did not occur with media from FGF11 siRNA-treated osteoclasts (Fig. 4f). FGF11 has been proposed to promote matrix metalloproteinase 9 (MMP9) signalling in T cells [28]. However, no effect of FGF11 siRNA was observed on hypoxic expression of either MMP9 mRNA (Fig. 4g) or protein (Fig. 4h).

**Fig. 5:**
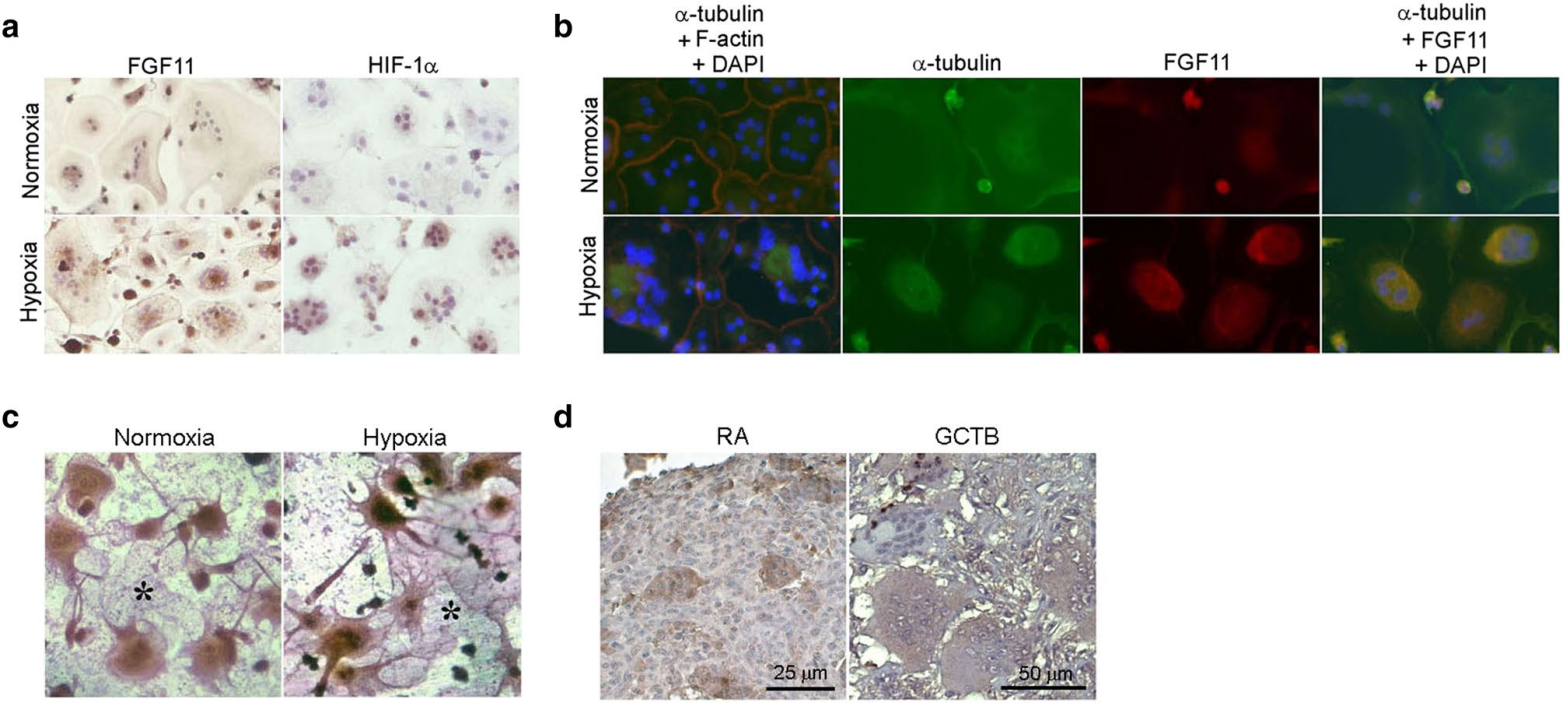
**a** Multi-nuclear mature human osteoclasts cultured on glass coverslips were exposed to normoxia or hypoxia (2% O_2_) for 24 h and stained for FGF11 or HIF-1α. **b** Mature osteoclasts cultured on glass coverslips were stained for total alpha-tubulin (*green*), F-actin (*red*) or FGF11 (*red*) following 24 h exposure to normoxia or hypoxia (2% O_2_). Nuclei were visualised with DAPI (4′,6-diamidino-2-phenylindole). **c** Actively resorbing FGF11-positive osteoclasts cultured on dentine discs reside above lacunar resorption pits (*single asterisk*). **d** FGF11 is strongly expressed by osteoclasts in representative images of the rheumatoid synovium (RA) and giant cell tumour of bone (GCTB)

As the available literature suggests a developmental or tissue remodelling role for FGF11 [19–22], we also examined the intracellular distribution of this FGF. Osteoclasts cultured on glass coverslips exhibited a diffuse FGF11 immunoreactivity in normoxia which intensified in the peri-nuclear region following hypoxic exposure (Fig. 5a). Hypoxic exposure was confirmed by staining for HIF-1α. This pattern of FGF11 immunostaining was reminiscent of the pattern of global alpha-tubulin staining that represents the microtubule arrangements observed in quiescent versus resorbing osteoclasts Lakkakorpi and Vaananen described microtubules in quiescent osteoclasts as being spread evenly over the whole cell, whereas those in resorbing osteoclasts were concentrated centrally over the resorption lacunae [29]. We also observed this pattern of alpha-tubulin staining in normoxic versus hypoxic osteoclasts (Fig. 5b). Fluorescent double staining for FGF11 and α-tubulin revealed co-localisation of the proteins in the central, peri-nuclear region of hypoxic osteoclasts (Fig. 5b). The normoxic signal for FGF11 was too weak to determine whether this co-localisation also occurs in normoxia. When FGF11 immunostaining was performed on osteoclasts cultured on dentine slices, the central concentration of FGF11 above resorption lacunae was clearly evident (Fig. 5c).

FGF11 was also expressed by the osteoclasts present in pathological conditions associated with excessive levels of osteoclast-mediated osteolysis. FGF11 expression was evident in large multinucleated cells morphologically resembling osteoclasts in representative cases of rheumatoid arthritis (RA) and giant cell tumour of bone (GCTB) (Fig. 5d).

## Discussion

Inhibition of osteoclast-mediated bone resorption is a therapeutic strategy to prevent pathological bone loss in conditions including rheumatoid arthritis, primary bone cancer, bone-metastatic cancer and osteoporosis. Over-expression of HIF-1α is a common feature of these conditions, associated with poor prognosis. Here, we describe FGF11 as a hypoxia- and HIF-1α-regulated gene in osteoclasts, which additionally drives hypoxic stimulation of osteoclast-mediated bone resorption.

Hypoxic induction of FGF11 mRNA and protein was observed in primary monocyte-derived osteoclasts and CD4+ T cells. FGF11 induction by PHD2 inhibition implied a role for HIF in this process, as was subsequently confirmed by HIF-1α siRNA. This is in agreement with Yang et al., who recently identified two HIF-1α-driven HREs in the FGF11 promoter in HUVEC [22]. However, it is noteworthy that FGF11 is generally not described in numerous studies performing genome-wide screening for HIF target genes [30–33]. Indeed, the only hit we found for FGF11 was reached using a computational strategy to combine transcriptional profiling meta-analysis of hypoxia-modulated genes with identification of HIF binding sites within potential cis-regulatory regions [34]. The reason for this is unclear but may indicate either a limited cell / tissue expression profile for FGF11 or reduced expression in the immortalised cell lines in which most screens are performed.

As hypoxic stimulation of osteoclast-mediated bone resorption is HIF-1α-dependent [10], we next sought to determine whether elevated expression of FGF11 could contribute to this response. siRNA targeting FGF11 significantly diminished the hypoxic increase in resorption. Conditioned media from hypoxic osteoclasts also stimulated bone resorption and this effect was also lost when the conditioned media was from FGF11 siRNA-treated osteoclasts. As FGF11 is an intracellular FGF, this must occur due to induction of a secreted factor downstream of FGF11. Few cytokines or growth factors have been described that can enhance bone resorption independent of effects on osteoclastogenesis. The main candidates are RANKL [35] and ANGPTL4 [11], however, RANKL is not produced by osteoclasts and ANGPTL4 expression was not modulated by FGF11. Hu et al. described FGF11-mediated stimulation of MMP9 production by prostate cancer cells [28]. However, the proposed mechanism involved secretion of FGF11 from infiltrating T cells, which should not occur. Regardless of the T cell / prostate cancer mechanism, MMP9 expression by osteoclasts was also not modulated by FGF11.

Although described as intracellular, it has been hypothesised that FGF11 could potentially be released from cells by a mechanism(s) independent of the endoplasmic reticulum and Golgi apparatus [14, 36]. FGF11 (and the other FHFs) does not possess a secretory signal sequence, but contains a nuclear localisation signal (NLS)-like sequence and is not secreted from transfected cell lines [36, 37]. However, these characteristics are shared with prototypical FGF1 and FGF2, which can be released from cells during cell migration [38] and via the Na+, K+-ATPase ion transporter [39]. These mechanisms are both directly relevant to osteoclast-mediated bone resorption, which relies heavily on both cell migration [40] and extracellular acidification by active transport of protons across the bone-apposing membrane [41]. However, we were unable to detect FGF11 in the conditioned media of either hypoxic osteoclasts or FGF11-transfected MG-63 cells by either Western blot or ELISA. It is possible that the concentration of secreted FGF11 was below the detection limit of our assays, in which case FGF11 itself might mediate the effects of hypoxic osteoclast conditioned media to stimulate bone resorption. However, there is currently no evidence available that this secretory mechanism(s) occurs for FGF11.

The small volume of literature available on FGF11 currently suggests a tissue remodelling function for this growth factor, with roles described in endothelial tube formation [22], odontogenesis [19] and skin wound healing [20, 21]. We therefore examined the intracellular distribution of FGF11 and observed the majority of FGF11 immunoreactivity in the central peri-nuclear region of the cell where it co-localised with α-tubulin, especially in actively resorbing hypoxic osteoclasts. This central concentration of microtubules over the resorption lacunae has been proposed to reflect the secretory function of resorbing osteoclasts [29]. Transcytotic vesicles containing the degraded bone matrix are associated with microtubules in bone-resorbing osteoclasts, suggesting that intracellular transport of transcytotic vesicles is supported by microtubules [42, 43]. It is therefore possible that FGF11 exerts its effect on bone resorption by interaction with α-tubulin or other microtubule-associated proteins. Alternatively, FGF11 might affect the expression or function of proteins that form the sealing zone in osteoclasts. Yang et al. reported FG11-mediated induction of the tight junction proteins occludin, ZO-1 and claudin-5 in HUVEC cells [22].

It is unfortunate that we were unable to identify a precise mechanism of action or a specific pathway activated downstream of FGF11. Limitations of the commercially available FGF11 antibodies precluded immunoprecipitation studies to determine whether FGF11 binds directly to α-tubulin or other microtubule-associated proteins. Additionally, the scale of screening we were able to perform was unable to identify either the secreted pro-resorptive factor induced by FGF11 or other intracellular signalling pathways activated by FGF11. However, it is hoped that our description of hypoxia-inducible, HIF-1α-regulated expression of FGF11 in osteoclasts and a role for FGF11 in mediating increased osteoclast-mediated bone resorption under hypoxic conditions will encourage further research on this intriguing growth factor.

The novel pro-resorptive function for FGF11 described here adds weight to the presumptive tissue remodelling role(s) of this growth factor. As it is also expressed in the osteoclasts associated with pathological bone resorption conditions such as rheumatoid arthritis, it represents a potential novel modulator of excessive osteolysis. Studies to further delineate the function(s) and signalling pathways involved in FGF11 biology may reveal exciting new therapeutic opportunities.
